# Kuttner’s Tumour: A Case Series and Narrative Review on Diagnosis, Management, and Outcomes

**DOI:** 10.3390/jcm14093208

**Published:** 2025-05-06

**Authors:** Zaid Al-Deerawi, Kamal El-Badawi, Arpan Shrivastava, Husham Barrak

**Affiliations:** 1 Queen Elizabeth Hospital, Birmingham B15 2GW, UK; 2 Queen Elizabeth the Queen Mother Hospital, Margate, Kent CT9 4AN, UK; kamal_el-badawi@live.com; 3 Ear, Nose, and Throat Department, Warwick Hospital, South Warwickshire University NHS Foundation Trust, Warwick CV34 5BW, UK; arpan.shrivastava@swft.nhs.uk (A.S.); hushamab@gmail.com (H.B.)

**Keywords:** Kuttner’s tumour, chronic sclerosing sialadenitis, submandibular gland, IgG4-related disease

## Abstract

**Background**: Kuttner’s tumour refers to chronic sclerosing sialadenitis commonly affecting the submandibular gland. It clinically mimics submandibular gland neoplasms and has a possible association with IgG4-related disease. The literature on standardised diagnostic and management pathways is limited. This paper presents a case series and narrative literature review to support a proposed diagnostic and management approach for Kuttner’s tumour. **Methods**: Twelve cases of patients with a Kuttner’s tumour aged between 55 and 87, identified from 2018 to 2025 through hospital records, were reviewed. All patients underwent ultrasound assessment using standardised diagnostic criteria and were followed up clinically and radiologically every six months. In parallel, we performed a narrative review of studies published between 2005 and 2025, identifying nine relevant articles to contextualise our findings. **Results**: Our 12-patient case series highlights the potential for Kuttner’s tumour to progress to bilateral involvement and IgG4-related disease. Most cases resolved spontaneously with ultrasound-led monitoring. One progressed to IgG4-RD and responded to glucocorticoids. Our findings support the selective use of invasive tests, baseline serum IgG4 testing, and a six-monthly follow-up strategy. Despite similarities within the existing literature, international variation highlights the need for standardised diagnostic and management protocols. **Conclusions**: We recommend a conservative, structured approach to managing Kuttner’s tumour, with six-monthly clinical and radiological follow-ups. Based on one case progressing to multi-organ IgG4-related disease, we now recommend routinely measuring serum IgG4 concentrations at diagnosis. The role of magnetic resonance imaging, fine-needle aspiration cytology, and biopsy should be considered on a case-by-case basis. Further research is needed to validate this approach and assess long-term outcomes.

## 1. Introduction

Kuttner’s tumour (KT) is a rare condition named after the German surgeon Hermann Kuttner who reported a series of four cases with unilateral submandibular gland swelling in 1896. KT refers to a chronic sclerosing sialadenitis usually affecting the submandibular gland (SMG) and can be classified into four stages histologically, with the end stage (stage IV) showing marked parenchymal loss and sclerosis. Patients with KT may report pain associated with food ingestion. The classic sign of KT is unilateral SMG swelling. Despite the name, it is a non-neoplastic condition and is not known to progress into malignancy. However, its presentation of a unilateral SMG swelling makes it an important mimic of neoplasia [1]. This is especially important as 22–50% of SMG tumours are malignant [2]. 

IgG4-related disease (IgG4-RD) is an inflammatory multi-organ condition that can affect any organ, including the head, neck, and salivary glands. IgG4-RD responds well to glucocorticoids. It has been suggested that there is an association between KT and IgG4-RD; however, this is controversial [3,4]. If left untreated, IgG4-RD can lead to end-stage organ failure and is associated with poor prognosis [5].

The existing literature on KT is scarce and there is no consensus on diagnostic tests and management. There are many reported approaches for the management of KT. Our centre’s approach relies on radiological ultrasound diagnosis and surveillance ultrasound scans every six months until the tumour has self-resolved. However, alternative approaches are described in the literature globally, including more invasive approaches such as fine-needle aspiration cytology (FNAC) and excision biopsy in centres where imaging alone is not considered sufficient for diagnosis.

KT has a similar presentation to SMG neoplasms and has a possible association with the potentially life threatening IgG4-RD. However, there are currently no guidelines in the United Kingdom (UK) to support diagnosis, treatment, and follow-up in patients with KT. Hence, the aim of this study is to present a case series of KT (with one case eventually being diagnosed with IgG4-RD) and explore the existing literature on KT in order to recommend a standardised approach for the diagnosis and management of this condition.

## 2. Methods

### 2.1. Case Series

The data for the case series were collected retrospectively from the hospital records of 12 patients over an eight-year period, between 2018 and 2025. The patients’ records were reviewed for initial clinical presentation, past medical history, imaging findings, treatment, and outcomes. Each case was assessed clinically in the head and neck clinic and evaluated with a neck ultrasound. Patients were followed up clinically and radiologically every six months. The diagnosis was made by the centre’s head and neck radiologist using the following diagnostic criteria:

- Well-demarcated geographical area of low echogenicity with increased vascularity;
- Mild focal intrinsic duct dilatation within the SMG;
- No suggestion of a neoplasm or space-occupying lesion.

### 2.2. Literature Review

A narrative review of the literature was conducted. This review follows a narrative/scoping approach and is not intended as a systematic review. The aim was to synthesise the most relevant literature on diagnostic and management pathways for KT, particularly in the context of IgG4-RD. We searched the following databases systematically: MEDLINE, PubMed, and Google Scholar. Our last search was conducted on 26 January 2025.

For the MEDLINE search, the terms used were: “Kuttner tumor.ti,ab.” and “chronic sclerosing sialadenitis AND submandibular gland.ti,ab.”, both excluding case reports using the filter “NOT case reports.pt.”. Similarly, PubMed was searched using the following terms: “kuttner tumor [Title/Abstract]”, “kuttner tumour [Title/Abstract]” and “chronic sclerosing sialadenitis in submandibular gland [Title/Abstract]”, all excluding case reports. On Google Scholar, we used the queries: “allintitle: kuttner tumor”, “allintitle: kuttner tumour” and “allintitle: chronic sclerosing sialadenitis in submandibular gland”, limiting the results by excluding case report entries via the “-Case -reports” operator in the title search.

Studies were included if they were full-text articles published in English between 2005 and 2025, involved human adult participants (>18 years), and addressed aspects of the diagnosis, management, or follow-up of KT. Studies were excluded if they were single case reports or animal studies.

**Figure 1:**
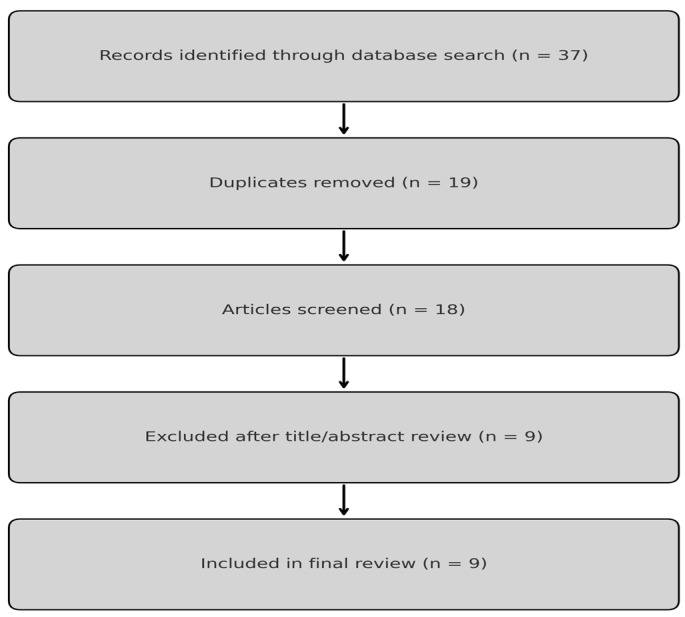
Article selection process for the narrative review. A total of 37 records were identified; after removing duplicates and screening for relevance, 9 articles were included in the final analysis.

The aim of this review was not to conduct a systematic or exhaustive analysis, but to summarise relevant studies that inform current clinical practice. Our search strategy identified a total of 37 articles. Removing duplicates left a total of 18 articles. Remaining articles were reviewed, of which 9 articles were found not to be relevant to our study’s aim. This left a total of 9 articles for the narrative review (Figure 1).

## 3. Results

**Figure 2:**
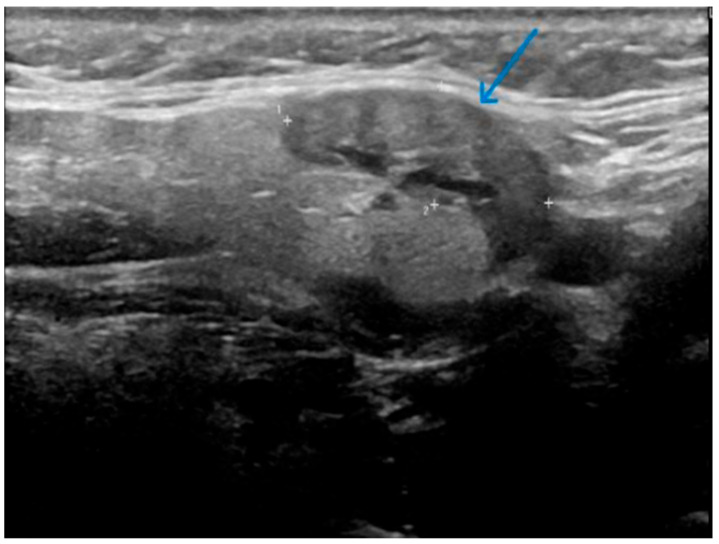
Patient X—Initial ultrasound scan of affected SMG. Well-defined, focal hypoechoic area (indicated by the blue arrow) in the left SMG with mild focal duct dilatation. The "+" markers represent measurement callipers to denote the borders of the hypoechoic lesion.

**Figure 3:**
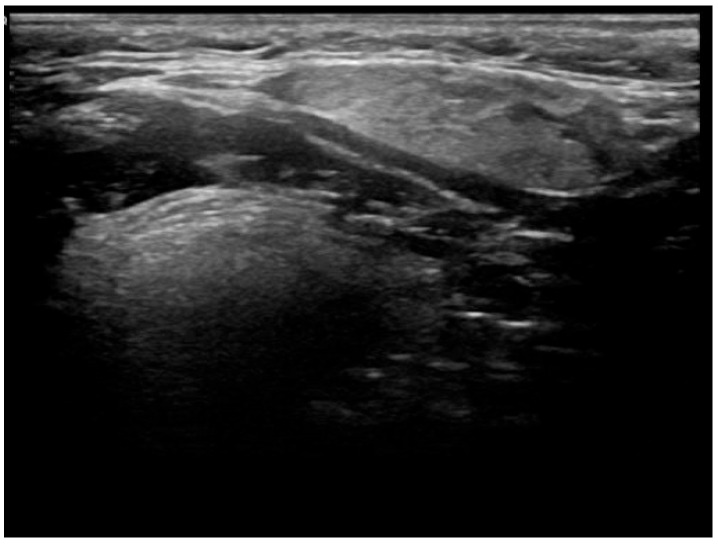
Patient X—Follow-up ultrasound scan of affected SMG. The previously identified abnormal area in the left SMG shows significant spontaneous resolution on follow-up imaging.

**Figure 4:**
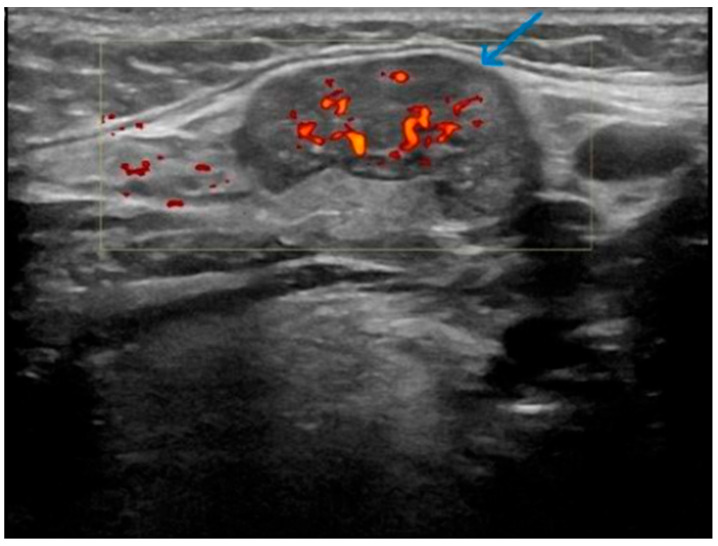
Patient Y—Initial ultrasound scan of affected SMG. Circumscribed focal area of low echogenicity and increased vascularity (indicated by the blue arrow) in the left SMG.

**Figure 5:**
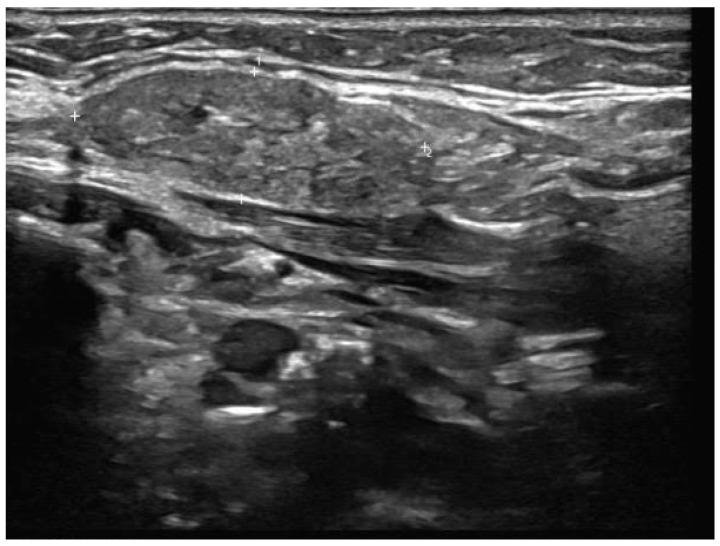
Patient Y—Follow-up ultrasound scan of affected SMG. Complete spontaneous resolution of previously identified lesion on follow-up imaging. The "+" markers represent measurement callipers.

**Figure 6:**
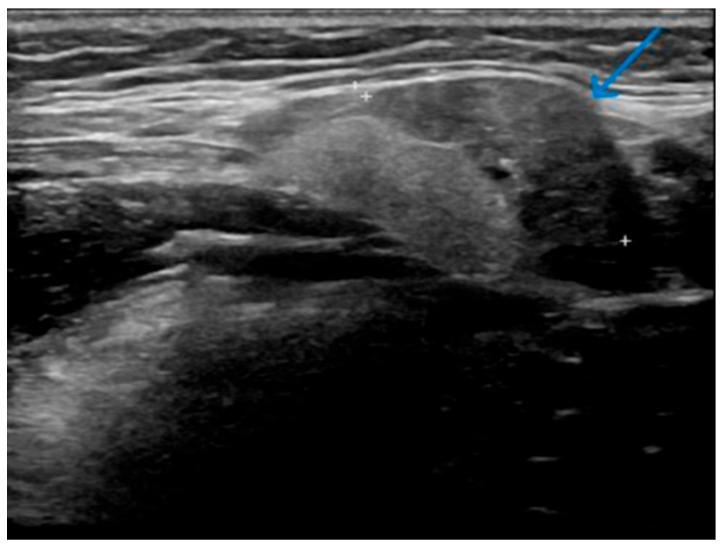
Patient Z—Initial ultrasound scan of affected SMG. Well-defined, focal hypoechoic area in the left SMG (indicated by the blue arrow) with mild focal duct dilatation. The "+" markers represent measurement callipers.

**Figure 7:**
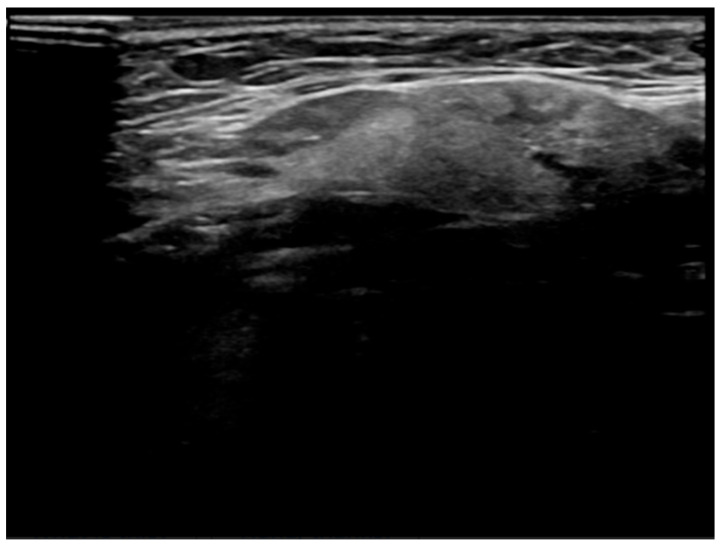
Patient Z—Follow-up ultrasound scan of affected SMG. The previously identified abnormal area in the left SMG shows significant spontaneous resolution on follow-up imaging.

This is a series of 12 cases of KT reviewed over an eight-year period, between 2018 and 2025 (Table 1). The age range is 55–87 years. The left SMG was the most commonly affected gland (8 out of the 12 cases on initial presentation), and the mean number of ultrasound follow-ups was 3.4. None of the patients had respiratory or sicca symptoms at initial presentation, and no history of inflammatory joint disease was reported. Based on clinical assessment, 11 cases showed complete resolution of symptoms and swelling. With regard to radiological findings, each of these cases showed spontaneous partial or complete resolution of the inflammatory changes in the SMG on follow-up ultrasound over a period ranging from 1 to 2 years [Figure 2, Figure 3, Figure 4, Figure 5, Figure 6 and Figure 7]. No patient in this group required immunosuppressive therapy. In this small cohort, there was no clear relationship observed between sex, laterality (left vs. right), or number of ultrasound follow-ups and time to recovery. For example, cases with both three and four follow-up scans showed recovery within the same 1.5–2 year range.

Out of the twelve cases, one case progressed to bilateral SMG involvement and subsequently to IgG4-RD. The disease initially presented as unilateral enlargement of the SMG, but in the follow-up appointment within 12 months progressed to bilateral disease. At this stage, the repeat ultrasound scan confirmed bilateral SMG involvement and, hence, the patient was referred to the rheumatology team who advised testing the serum IgG4 concentration, which returned as positive. The patient was referred to the rheumatology department and managed using a multidisciplinary team approach. The patient was followed up by the rheumatology department and developed the symptoms of dry eyes and dry mouth, and was found to have kidney involvement (i.e., multi-organ disease). The patient was treated with glucocorticoids and continues to be followed up by rheumatology.

This suggests that bilateral submandibular gland disease may be associated with a higher risk of underlying IgG4-RD, but the size of the sample is too small to draw definitive conclusions. While the numbers are too small for statistical testing, this observational comparison may inform future study design and highlights the potential value of routine IgG4 concentration testing and bilateral gland surveillance at baseline. This case has prompted a change in practice at our centre, with routine serum IgG4 concentration testing now recommended at diagnosis of KT to facilitate earlier identification of systemic disease. This may enable earlier rheumatology input and pre-empt multi-organ involvement.

## 4. Discussion

### 4.1. Overview and Contribution to the Literature

Our case series offers a unique contribution to the limited UK-based literature on KT, particularly regarding long-term follow-up and conservative management. Only four of the nine articles identified in our literature search were published in the last decade. More recent studies, including those by Muniz et al. and Peuraharju et al., introduced significant developments in disease management while also highlighting the limited availability of data [6,7]. While earlier works such as those by Kitagawa et al., Chow et al., and Machado De Sousa et al. played a crucial role in establishing recognition of the disease entity [8,9,10], they did not provide clear guidance on management at an international level. Culver et al. remains the only directly comparable UK study, but it also did not outline detailed guidance on follow-up protocols or diagnostic methods [11].

The existing literature consists almost entirely of retrospective data with varying sample sizes, ranging from as few as two cases (Machado De Sousa et al.) to a maximum of fifty-one (Furukawa et al.) [10,12]. Only a limited number of studies explicitly identified cases that had developed IgG4-RD. Muniz et al. and Geyer et al. each reported three cases of KT associated with IgG4-RD [3,6]. This highlights the rarity of such progression and the need for larger, standardised datasets to establish robust diagnostic and management pathways. A summary of the key studies identified during the literature review is provided in Table 2.

### 4.2. Diagnostic Approach and Imaging

In our practice, routine serum IgG4 testing at baseline may have facilitated earlier detection in the single case that progressed to IgG4-RD. However, the role of serum concentrations of IgG4 in diagnosing IgG4-RD is debated in the literature. While serum IgG4 concentrations are not considered the gold standard for detecting IgG4-RD, Peuraharju et al. highlighted their importance in certain cases [7]. They suggested that serum IgG4 testing should be reserved for patients with clinical manifestations beyond the salivary glands or where IgG4 positivity has been detected in tissue specimens. Furukawa et al. and Culver et al. also emphasised the use of serum IgG4 concentrations in conjunction with histopathological findings from SMG biopsies [11,12]. Although serum IgG4 concentrations are not considered a standalone diagnostic marker, our experience suggests that incorporating them into the initial assessment may raise early clinical suspicion, particularly in cases with bilateral disease or atypical features.

All patients in our series were assessed using neck ultrasound, and the diagnosis of KT was made by our head and neck radiologist using standardised sonographic criteria. Peuraharju et al. reported that all but two of their patients underwent a diagnostic ultrasound, with alternative imaging performed due to the presence of an intraglandular mass or sialolith [7]. Culver et al. highlighted ultrasound’s ability to detect glandular and lymph node enlargement, emphasising the importance of ultrasound-guided biopsy in diagnosis [11]. Uhliarova et al. also underscored ultrasound’s value as an initial investigation tool [13]. However, Chow et al. did not report why some patients in their cohort did not undergo a preoperative ultrasound, leading to inconsistencies in diagnostic pathways. Furukawa et al. noted that when combined with diagnostic criteria, ultrasound could help identify IgG4-related dacryoadenitis and sialadenitis [12]. However, this is not specific to KT. This is in contrast with our practice, which supports ultrasound as an easily accessible and cost-effective diagnostic tool. The study by Uhliarova et al. was the only one to formally include ultrasound as part of the diagnostic pathway [13]. While no difference in patient outcomes was noted, the variation in practice highlights the need for greater consistency in clinical decision-making. Our experience suggests that ultrasound can be sufficient in typical cases, reducing the need for more invasive testing. However, this is only reliable when the ultrasound scan is performed and interpreted by a head and neck radiologist experienced in KT. Additional imaging (and invasive tests) should be considered when atypical features are present.

Whilst Magnetic Resonance Imaging (MRI) is not routinely used in our practice, its use varies in the literature. The diagnostic framework proposed by Culver et al. included the recommendation of MRI for patients with features of systemic disease or high serum IgG4 levels, alongside ultrasound-guided biopsy [11]. MRI assesses disease distribution and differentiates KT from other conditions such as Sjogren’s syndrome and sarcoidosis. Additionally, it aids in detecting stones in the Wharton’s duct, as noted by Chow et al. [9]. While our patients were effectively diagnosed and followed up with using ultrasound alone, MRI might be a valuable adjunct in patients with suspected systemic involvement or diagnostic uncertainty.

In radiologically confirmed KT without ultrasound features concerning for malignancy, we avoid invasive testing such as FNAC. We do consider FNAC in cases where there are clinical or radiological features suggestive of malignancy or in any cases of disease progression. Literature highlights the widespread use of FNAC, being a simple, safe, and cost-effective technique for diagnosing salivary gland masses, with high sensitivity and specificity [14]. However, details on its use in KT are limited in the literature. Uhliarova et al. reported that most patients in their cohort underwent FNAC, except for two who had stones detected during imaging [13]. FNAC provided valuable cytological information to determine the aetiology of glandular swelling. However, there is a risk of obtaining an inadequate sample with FNAC, which may delay the diagnostic process. Similarly, Chow et al. performed FNAC on most of their cohort, except for those with stones [9]. When FNAC is combined with ultrasound, it proves useful in distinguishing KT from neoplasms but was not effective in identifying IgG4-RD [9]. Our experience suggests that FNAC may be unnecessary in typical cases of KT when sonographic features are consistent and there is no suspicion of malignancy. Avoiding FNAC in these scenarios may streamline the diagnostic process while minimising patient burden. However, FNAC retains value in cases with suggestions of malignancy.

A key difference between our practice and the existing literature is the role of excision in managing SMG swelling. Excision was not part of our standard pathway in cases of suspected KT. The literature provides limited data on the benefits of excision, as nearly all cases underwent surgical removal for histological analysis, with only one case managed via an incisional biopsy. Nevertheless, discussions and recommendations have shaped current management approaches. Uhliarova et al. suggested that KT can be managed conservatively in asymptomatic patients, though excision is often performed due to the difficulty of distinguishing it from a neoplasm [13]. Culver et al. supported this view, recommending ultrasound-guided biopsy of the SMG in suspected KT associated with IgG4-RD [11]. However, with advances in imaging and immunohistochemistry, routine excision may not always be necessary. Peuraharju et al. highlighted the risks associated with excision of suspected neoplastic lesions, including an increased risk of tumour recurrence [7]. Open excisional biopsies of SMG lesions also carry the risk of nerve injury. Therefore, we advocate for a more selective approach: while excision remains important for definitive diagnosis in uncertain or atypical cases, routine surgical excision may not be justified, especially given the associated morbidity and the absence of differences in outcome compared to conservative or minimally invasive management.

In our series, only one case progressed to IgG4-RD. A number of different diagnostic frameworks (such as those proposed by Umehara et al. and the Boston Consensus Statement) offer varied criteria based on the debated specificity and sensitivity of serum IgG4 concentrations [15,16]. There remains no general consensus in the existing literature regarding the most appropriate definition criteria for IgG4-RD. Muniz et al. and Furukawa et al. used the Umehara et al. criteria to define IgG4-RD, whereas Peuraharju et al. and Culver et al. followed the Boston Consensus Statement [6,7,11,12].

The 2020 revised comprehensive diagnostic (RCD) criteria by Umehara et al. incorporates serum IgG4 concentrations as part of the disease definition, allowing them to be used alongside histological, clinical, and radiological findings [17]. In contrast, the Boston Consensus Statement, cited by Peuraharju et al. [7] and Culver et al. [11], focuses on immunohistochemistry and histopathological analyses of biopsy samples without considering serum IgG4 levels [15]. The latter approach may leave room for systemic disease to go undetected, despite elevated IgG4 levels in SMG biopsies [15]. Interestingly, studies that applied the 2020 revised RCD criteria for IgG4-RD exclusively identified cases of true chronic sclerosing sialadenitis, whereas cases identified by Peuraharju et al. were non-sclerosing. Given these data, it is difficult to firmly support the Boston Consensus Statement as the preferred method for identifying KT associated with IgG4-RD. If serum IgG4 is available at presentation, incorporating Umehara et al.’s criteria may aid earlier clinical suspicion and guide further investigation. In contrast, the Boston Consensus Statement’s reliance on histology may delay diagnosis. A flexible approach combining both frameworks may be most effective until a consensus is reached.

### 4.3. Management Strategies and Follow-Up

In our single case that progressed to IgG4-RD, treatment with glucocorticoids showed a clear clinical benefit. However, the small number of cases in the literature and reliance on excisional biopsy as the primary diagnostic tool limited the ability to draw broader conclusions. Our findings reflect a wider issue in the literature: the absence of standardised management protocols and over-reliance on surgical intervention for diagnosis. Muniz et al., who reported the largest sample of cases with IgG4-RD, did not present clinical data related to management [6]. Peuraharju et al. described two cases of IgG4-RD exhibiting non-sclerosing chronic sialadenitis [7]. One patient, with mildly elevated serum IgG4 concentrations, underwent SMG resection followed by a seven-month course of prednisolone and remained symptom-free at follow-up. Another patient, with significantly raised serum IgG4 levels, also underwent SMG resection followed by oral prednisolone, with azathioprine added later. Despite this treatment, the patient developed persistent lung symptoms and contralateral SMG involvement after two years, though no further outcomes were reported. Furukawa et al. similarly documented the resolution of SMG swelling after excision and steroid treatment, although follow-up details were limited [12]. Importantly, we observed no relapse in our single IgG4-RD case treated with glucocorticoids, though long-term monitoring is ongoing. Geyer et al. reported a patient with multiorgan IgG4-RD who achieved long-term remission with glucocorticoids, and another with progressive disease involving multiple glands and organs, though incomplete data limited interpretation [3]. Kitagawa et al. reported a single case where biopsy findings suggested chronic sclerosing sialadenitis with IgG4-RD, indicated by IgG4-related sclerosing inflammation in the SMG [8]. This patient experienced spontaneous regression without medical or surgical treatment, suggesting conservative management may be viable in select cases. Culver et al., while not reporting on individual cases, emphasised the value of multidisciplinary care and highlighted the risk of relapse upon steroid withdrawal [11]. Our experience and the published evidence both agree on the benefit of using glucocorticoids. While some patients benefited from surgery followed by immunosuppression, others experienced a relapse or systemic progression, highlighting the importance of multidisciplinary management. Conservative approaches may be appropriate for asymptomatic or stable disease, with longer-term data needed. Ultimately, a unified diagnostic and treatment framework is essential to optimise outcomes and reduce unnecessary surgical interventions.

Our local standardised approach for surveillance methodology and frequency is six-monthly clinical and radiological monitoring. Helping to balance the clinical necessity of early detection of disease progression with the practicality of routine follow-up in stable patients. The existing literature provides limited data on surveillance methods for KT, particularly in cases associated with IgG4-RD. Geyer et al. referenced the use of radiological monitoring, suggesting that the systemic nature of the disease in their cases warranted CT or MRI, though ultrasound was not mentioned and follow-up frequency was unspecified [3]. Uhliarova et al. highlighted the benefits of frequent monitoring for patients with KT, citing Fiorella et al., who recommended follow-up intervals every 2–3 months initially, then every 6–12 months, with annual ultrasound for benign salivary gland lesions [13,18].However, serum IgG4 monitoring is not included in this approach and therefore it does not account for the risk of IgG4-RD progression. Furukawa et al. mentioned the use of clinical or radiological monitoring during follow-up but did not specify further follow-up methods or frequency [12]. Peuraharju et al. were the only study group to use a symptom questionnaire to track recurrence, identifying symptoms up to two years after the initial presentation [7]. However, the lack of radiological monitoring meant that disease progression could not be assessed. Additionally, this approach had limitations, including response rates and language barriers. Kitagawa et al. reported a case of KT with suspected IgG4-RD that underwent biopsy, but no follow-up duration or monitoring methods was detailed [8].

In our series, ultrasound played a key role in both diagnosis and monitoring. Typical ultrasound findings in KT show diffuse involvement of the gland; however, focal lesions have also been described [19]. At our centre, most cases demonstrated localised involvement of the SMG. Our standardised follow-up strategy of biannual clinical and ultrasound monitoring proved effective in detecting both resolution and progression, as demonstrated by our single case that evolved from presumed unilateral KT to bilateral disease and ultimately to IgG4-RD. Regular imaging enabled timely identification of this progression, prompting early referral to rheumatology and initiation of immunosuppressive therapy. For the remaining 11 patients, structured follow-up confirmed spontaneous resolution without the need for intervention. This experience highlights the value of a consistent, non-invasive surveillance protocol in real-world practice. Compared to the fragmented strategies in the literature, our approach offers a practical framework that balances vigilance with efficiency.

## 5. Conclusions

The existing literature highlights a lack of consensus on both the diagnosis and management of Kuttner’s tumour, particularly in cases associated with IgG4-RD. Our recorded case of Kuttner’s tumour progressing into multi-organ IgG4-RD is a signal to improve our centre’s current approach to patients with Kuttner’s tumour. We recommend that every patient diagnosed with Kuttner’s tumour to have their serum IgG4 concentrations tested. In addition, we recommend a six-monthly clinical and radiological monitoring. MRI, FNAC, and biopsy are controversial and should be case-dependent. Further research is needed to explore the outcomes of patients with Kuttner’s tumours managed with our recommendations.

## Figures and Tables

**Table 1:** Summary of clinical characteristics, investigations, follow-up, and outcomes of the case series.

Case	Sex	SMG Affected	Symptoms of Dry Eye and Dry Mouth at Initial Presentation	Respiratory Symptoms at Initial Presentation (Cough or Shortness of Breath)	Past Medical History of Inflammatory Joint Disease at Initial Presentation	IgG4 Concentrations Tested	Number of Follow-Up Ultrasounds	Outcome	Time from Diagnosis Until Recovery (Years)	Immunosuppressive Therapy Required
1	Female	Left	No	No	No	No	3	Resolved	1.5	No
2	Female	Left	No	No	No	No	4	Resolved	2	No
3	Male	Left	No	No	No	No	4	Resolved	2	No
4	Female	Right	No	No	No	No	3	Resolved	1.5	No
5	Male	Right	No	No	No	No	3	Resolved	1.5	No
6	Female	Left	No	No	No	No	3	Resolved	1.5	No
7	Female	Left	No	No	No	No	3	Resolved	1.5	No
8	Female	Left	No	No	No	No	3	Resolved	1.5	No
9	Male	Left	No	No	No	No	3	Resolved	1.5	Yes
10	Female	Left	No	No	No	No	4	Resolved	2	
11	Male	Right	No	No	No	No	4	Resolved	2	
12	Female	Right then left	No	No	No	Yes	4	IgG4-RD	Change to IgG4-RD	

**Table 2:** Literature review summary table.

Article	Date of Publication	Country of Publication	Sample Size	Cases with Presence of IGg4 Related Disease	Investigation	Igg4 Definition Criteria	Management of Kuttner’s Tumour With IgG4-Related Disease	Surveillance Method and Frequency
Muniz et al. [6].	2024	Brazil	17	3	Excisional biopsy Immunohistochemistry Histopathology	Umehara et al.	Not reported	Not reported
Peuraharju et al. [7].	2020	Finland	51	2 (both classified as non-sclerosing chronic sialadenitis. One of these cases had a preceding IgG4 related dacryoadenitis)	Excisional biopsy Immunohistochemistry Histopathology Preoperative ultrasound (*n* = 49) MRI (*n* = 9) CT (*n* = 2) Serum IgG4 level (*n* = 2)	Boston consensus statement	Oral prednisolone 7 months (*n* = 1) Oral prednisolone and azathioprine (*n* = 1)	Symptom questionnaire
Furukawa et al. [12].	2015	Japan	54	1	Excisional biopsy Immunohistochemistry Histopathology Serum IgG4 level (*n* = 6)	Umehara et al.	Oral prednisolone (*n* = 1)	Recurrence documented, method of monitoring not reported
Culver et al. [11].	2015	UK	N/A	N/A	Serum IgG4 level USS head and neck Fine-needle aspiration Ultrasound guided biopsy Excisional biopsy Immunohistochemistry Histopathology	Boston consensus statement	N/A	Recognised risk of recurrence, no method of monitoring suggested
Uhliarova et al. [13].	2013	Slovakia	7	0	Pre-operative ultrasound (*n* = 7) CT (*n* = 2) Fine-needle aspiration (*n* = 5)	Not applicable	Not applicable	Mention of annual USS to monitor recurrence of CSS, nothing specific to IgG4 related disease
Geyer et al. [3].	2010	USA	13	3	Serum IgG4 levels (*n* = 11) Biopsy [unspecified] (*n* = 2) CT (*n* = 1)	Clinical/pathological evidence	Glucocorticoids (*n* = 1)	Follow-up method not specified (mean follow-up of 6 years)
Machado De Sousa et al. [10].	2008	Brazil	2	0	Sialogram (*n* = 2) CT (*n* = 1)	Not reported	Not applicable	Not reported
Chow et al. [9].	2008	Hong Kong	9	0	USS (*n* = 9) CT (*n* = 2) MRI (*n* = 1) Fine-needle aspiration and cytology (*n* = 6)	Not applicable	Not applicable	Not reported
Kitagawa et al. [8].	2005	Japan	12	Not formally identified: 45% of IgG-positive plasma cells were of IgG4-type in all CSS cases	Not reported	Not applicable	Not applicable (all samples underwent excisional biopsy)	Not reported

Abbreviations: N/A, not applicable.

## Data Availability

The data presented in this study are available on request from the corresponding author.

## Abbreviations

The following abbreviations are used in this manuscript:

CT	Computer Tomography
FNAC	Fine-Needle Aspiration Cytology
IgG4-RD	Immunoglobulin-Related Disease
KT	Kuttner’s Tumour
MEDLINE	Medical Literature Analysis and Retrieval System Online
MRI	Magnetic Resonance Imaging
RCD	Revised Comprehensive Diagnostic
SMG	Submandibular Gland
UK	United Kingdom
N/A	Not Applicable
